# Chronic stress induces Alzheimer’s disease-like pathologies through DNA damage-Chk1-CIP2A signaling

**DOI:** 10.18632/aging.205862

**Published:** 2024-05-30

**Authors:** Zhuoqun Wang, Lun Zhang, Jiayu Yang, Yi Zeng, Chengke Su, Mengdong Yao, Huiliang Zhang, Wenting Hu, Yi Liu, Yiwen Lai, Xiaochuan Wang, Ji Zeng, Rong Liu

**Affiliations:** 1 Department of Pathophysiology, Key Laboratory of Ministry of Education/Hubei Province for Neurological Disorders, School of Basic Medicine, Tongji Medical College, Huazhong University of Science and Technology, Wuhan, China; 2 Department of Clinical Laboratory, Wuhan Fourth Hospital, Wuhan, China; 3 Shenzhen Huazhong University of Science and Technology Research Institute, Shenzhen, China; 4 Department of Pediatrics, Tongji Hospital, Tongji Medical College, Huazhong University of Science and Technology, Wuhan, China; 5 Institute for Brain Research, Wuhan Center of Brain Science, Huazhong University of Science and Technology, Wuhan, China; 6 Department of Pathology, Peking University Shenzhen Hospital, Shenzhen, China

**Keywords:** stress, Alzheimer’s disease, DNA damage, Chk1, CIP2A

## Abstract

Stress is an important initiating factor in promoting Alzheimer’s disease (AD) pathogenesis. However, the mechanism by which stress induces AD-like cognitive impairment remains to be clarified. Here, we demonstrate that DNA damage is increased in stress hormone Corticotropin-releasing factor (CRF)-treated cells and in brains of mice exposed to chronic restraint stress. Accumulation of DNA damage drives activation of cell cycle checkpoint protein kinase 1 (Chk1), upregulation of cancerous inhibitor of PP2A (CIP2A), tau hyperphosphorylation, and Aβ overproduction, eventually resulting in synaptic impairment and cognitive deficits. Pharmacological intervention targeting Chk1 by specific inhibitor and DNA damage by vitamin C, suppress DNA damage-Chk1-CIP2A signaling pathway in chronic stress animal model, which in turn attenuate AD-like pathologies, synaptic impairments and cognitive deficits. Our study uncovers a novel molecular mechanism of stress-induced AD-like pathologies and provides effective preventive and therapeutic strategies targeting this signaling pathway.

## INTRODUCTION

Stress response is a physiological reaction to adverse life events that interfere the ability to handle and adapt, and the adverse life events are also known as stressors [1]. Monoamines, including noradrenaline (NA), dopamine (DA), and serotonin (ST) are released rapidly during the first stage of stress response. The hypothalamus-pituitary-adrenal (HPA) axis is also activated in response to stressors subsequently. Corticotropin-releasing factor (CRF), vasopressin and adrenocorticotropic hormone (ACTH) are released in brain. Then ACTH promotes the synthesis and release of glucocorticoid hormones (GC) from the adrenal cortex, the latter, induces functional changes of multiple organs to meet the body needs upon the challenge of stressors [2, 3]. Besides inducing stress response in peripheral organs, stress hormones also manipulate the function of neuronal cells directly. Stress may be positive, it could help human or animal adapt to changes in the environment, even enhance cognitive function. However, persist and strong stress response is harmful and contributes to the onset and development of disorders in various systems and organs, including mental disorders such as depression, posttraumatic stress disorder (PTSD), sleep disturbance and Alzheimer’s disease (AD) [4–9].

Epidemiological investigation in humans and studies in animal models indicate that chronic stress might be an early event in the development of AD. Chronic stress may precede preclinical AD, which manifests as subjective cognitive decline (SCD) [10]. Frequent exposure to chronic stress is associated with the development of AD [11–13]. In addition to epidemiological evidences, some animal experiments also show the role of stress in promoting AD and partially elucidate the possible mechanisms. It has been reported that glucocorticoids could induce tau hyperphosphorylation by activation of glycogen synthase kinase-3 (GSK-3β) [14] and downregulate the expression brain-derived neurotrophic factor (BDNF) [15]. These events eventually evoked synaptic weakening and cognitive impairment. Another research identified that chronic restraint stress (CRS) promoted inhibition of protein phosphatase 2A (PP2A), which is the key phosphatase participating in tau dephosphorylation, thus causing tau hyperphosphorylation and cognitive dysfunction in mice [16]. In general, both epidemiologic and laboratory evidences suggested that stress is a potential causal factor of dementia. However, the mechanism by which stress promotes the development of AD has not been fully elucidated, and there are also no effective targets for early prevention and treatment.

High-level of DNA damage in the brain and plasma was found in patients suffering from depression [17, 18]. In addition, elevated DNA oxidative damage was also detected in hippocampus or frontal cortex of animals exposed to different types of chronic stress [19–21]. Meanwhile, DNA damage is an early event during the progression of AD. γH2A.X, a DNA double-strand breaks (DSB) marker, was upregulated in neurons and astrocytes in the brain of mild cognitive impairment (MCI) and AD patients [22]. In our previous study, we have demonstrated that DNA damage was accumulated in primary neurons treated with Aβ and brain tissues of APP/PS1 mice [23]. Therefore, we speculate that stress may promote the development of AD pathology through signaling pathway initiated from DNA damage. Our previous research has been confirmed that overexpression of cancerous inhibitor of PP2A (CIP2A), an endogenous PP2A inhibitor, could induce tau hyperphosphorylation and Aβ overproduction, eventually lead to synaptic degeneration and cognitive deficits [24]. Moreover, DNA damage could activate cell cycle checkpoint protein kinase 1 (Chk1), the upstream regulator of CIP2A, and induce AD-like pathologies in animal brains and cells [23].

Based on these findings, we suspect that stress may contribute to the occurrence and development of AD through the activation of DNA damage-Chk1-CIP2A signaling pathway. In this study, we report that accumulation of DNA damage, activation of Chk1-CIP2A signaling pathway and AD-like pathology appear in cell and animal stress models. And vitamin C and Chk1 inhibitor can rescue these pathologic changes effectively.

## RESULTS

### Acute and chronic stress induce DNA damage in animal models

**Figure 1:**
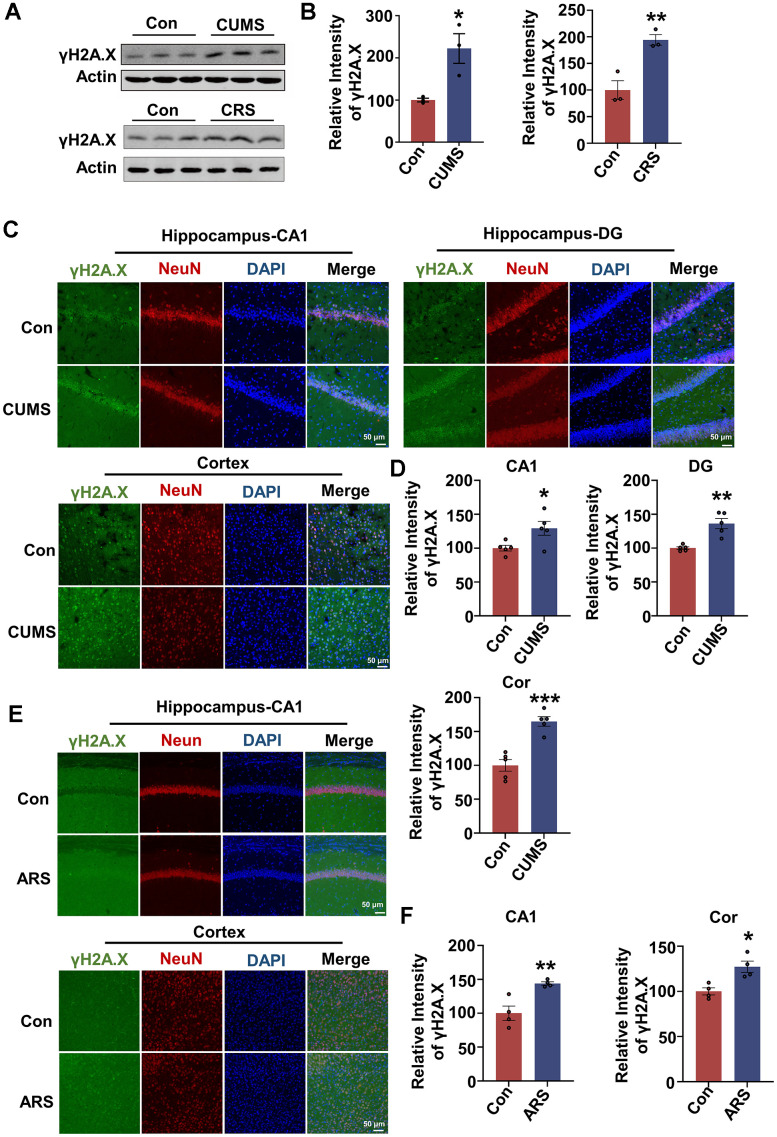
**Acute and chronic stress induce DNA damage in animal models.** (**A**) Representative immunoblots of γH2A.X and β-actin in hippocampal tissues from chronic unpredictable mild stress (CUMS), chronic restraint stress (CRS) model and control animals. (**B**) Quantification of the relative protein levels of γH2A.X, which were normalized to the β-actin levels. n = 3 per group. (**C**) Representative fluorescence images of γH2A.X (green), NeuN (red) and DAPI (blue) in hippocampus (CA1 and DG region) and cortex in CUMS rats. Scale bar: 50 μm. (**D**) Quantitative analysis of the fluorescence intensity of γH2A.X in C. n = 5 per group. (**E**) Representative fluorescence images of γH2A.X (green), NeuN(red) and DAPI (blue) in hippocampal CA1 region and cortex in acute restraint stress (ARS) mice. Scale bar: 50 μm. (**F**) Quantitative analysis of the fluorescence intensity of γH2A.X in E. n = 4 per group. Data are presented as mean ± SEM. *P < 0.05, **P < 0.01, ***P < 0.001.

To explore whether DNA damage occurs under different stress conditions, we established three stress animal models. Chronic unpredictable mild stress (CUMS) model is a reliable chronic stress model, rats are randomly stimulated with different stressors to induce anxiety- and depression-like behaviors [25]. Another common chronic stress model is chronic restraint stress (CRS) model. In this model, mice are restrained movements for 3 hours per day for 21 consecutive days. It represents a predictable, mild and effective stress model. At last, another group of mice are limited movements for 3 hours just once, which is used to induce acute restraint stress (ARS) with physical and emotional dysfunction temporarily [26]. We observed significant DNA damage in the brain of CUMS rat model (Figure 1A–1D), which was manifested by increased γH2A.X level in hippocampus (Figure 1A, 1B). The DNA damage was prominent in hippocampal CA1, DG and brain cortex, and mainly occurred in neurons, manifested by increased immunostaining of γH2A.X in NeuN-positive cells, not in GFAP-positive or Iba1-positive cells (Figure 1C, 1D and Supplementary Figure 1A–1C). Similarly, DNA damage was observed in the hippocampus of the CRS model (Figure 1A, 1B). In addition to chronic stress, the acute restraint stress also caused DNA damage. Compared to control, increased DNA damage occurred in hippocampal CA1 region and cortex of ARS model, not in hippocampal CA3 and DG regions (Figure 1E, 1F). These data collectively indicate that stress induces DNA damage, and neurons in CA1 region and brain cortex may be more susceptible to DNA damage than those in other regions.

### Stress hormone causes DNA damage, activates Chk1-CIP2A pathway, results in tau hyperphosphorylation, Aβ overproduction and synaptic impairments in primary neurons

**Figure 2:**
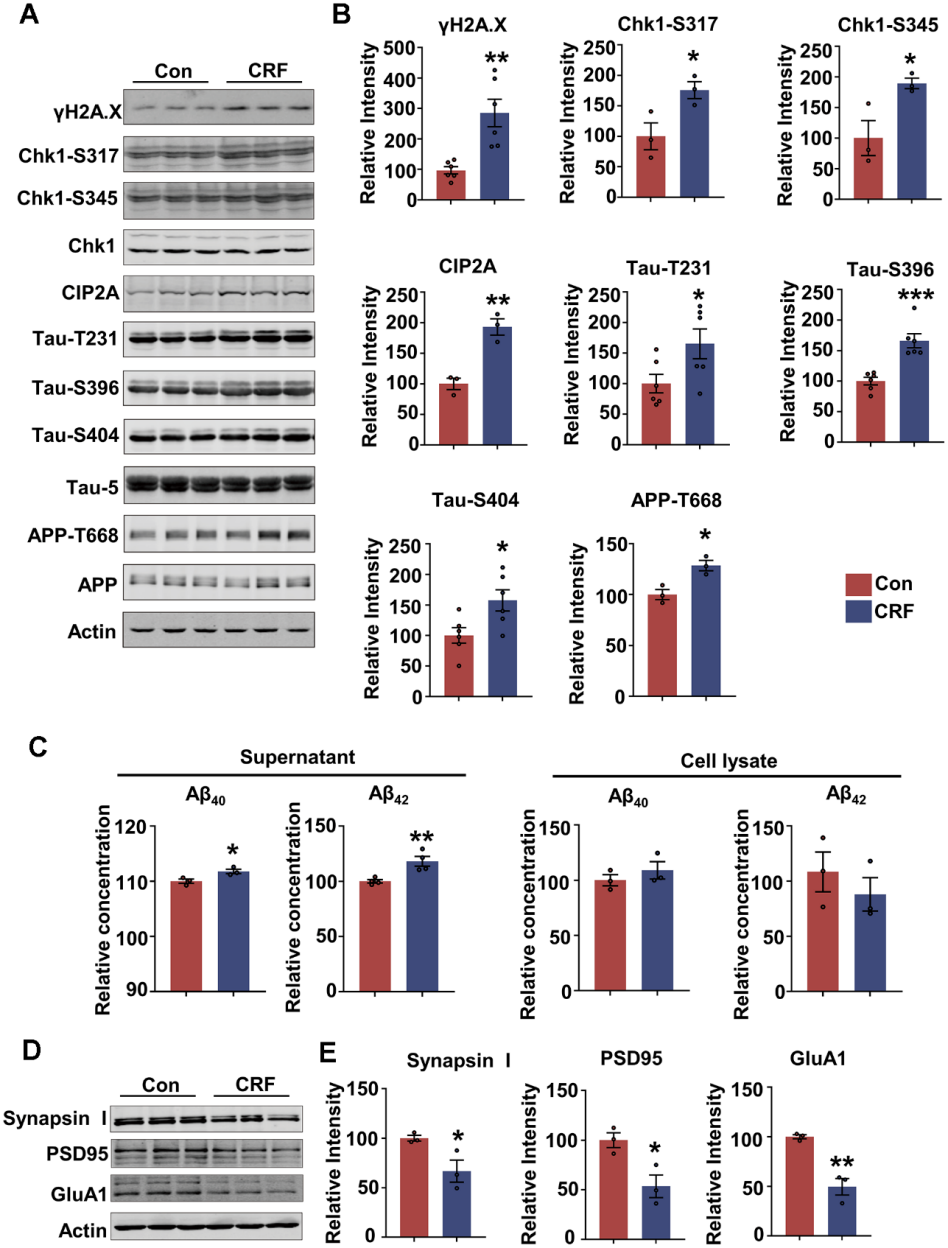
**Stress hormone activates DNA damage-Chk1-CIP2A pathway, results in tau hyperphosphorylation, Aβ overproduction and synaptic impairments in primary neurons.** (**A**–**E**) Primary neurons were treated with 100 nM corticotropin-releasing factor (CRF) for 24 hours. (**A**) Representative immunoblots of γH2A.X, Chk1-pS317, Chk1-pS345, Chk1, CIP2A, Tau-pT231, Tau-pS396, Tau-pS404, Tau-5 (total tau), APP-pT668, APP and β-actin. (**B**) Quantification of the relative protein levels; non-phosphorylated proteins such as γH2A.X and CIP2A were normalized to the β-actin levels; phosphorylated Chk1-pS317, Chk1-pS345, Tau-pT231, Tau-pS396, Tau-pS404 and APP-pT668 were normalized to corresponding total Chk1, tau (Tau-5) and APP respectively. n = 3 or n = 6 per group. (**C**) The relative concentration of Aβ_40_ and Aβ_42_ in supernatant and cell lysate of primary neurons detected by ELISA. n = 3 or n = 4 per group. (**D**) Representative immunoblots of Synapsin I, PSD95, GluA1 and β-actin. (**E**) Quantification of the relative protein levels, which were normalized to the β-actin levels. n = 3 per group. Data are presented as mean ± SEM. *P < 0.05, **P < 0.01, ***P < 0.001.

To mimic chronic stress in primary neurons, and evaluate whether AD-related pathologies appear in stress condition and its potential mechanisms, we used two stress hormones, corticosterone (CORT) and corticotropin-releasing factor (CRF) to treat primary neurons. The results showed that the protein level of γH2A.X was increased in dose-dependent manner in primary neurons treated with CORT and CRF for 24 hours (Supplementary Figure 2B, 2D). Meanwhile, LDH assay showed 300 or 400 μM CORT which induced DNA damage also caused significant cytotoxicity, whereas 50 nM or 100 nM CRF did not induce significant cell death (Supplementary Figure 2A, 2C). In addition, we attempted to treat primary neurons with lower dose of CORT (10 or 50 μM) for 48 hours. However, 50 μM CORT induced cytotoxicity but no DNA damage (Supplementary Figure 2A, 2B). Thus, we used CRF to treat primary neurons to mimic the chronic damage induced by stress. While 50 nM CRF did not cause tau hyperphosphorylation at Thr231 and Ser396, it was less stable than 100 nM CRF in causing AD-like pathologies. So, we finally chose primary neurons treated with 100 nM CRF for 24 hours as the chronic stress cell model. We found that besides causing DNA damage, 100 nM CRF incubation induced tau hyperphosphorylation (at Thr231, Ser396 and Ser404) and phosphorylation of APP at Thr668 in primary neurons (Figure 2A, 2B). Moreover, Aβ_40_ and Aβ_42_ levels were increased in culture media of neurons after CRF treatment, suggesting that CRF could induce Aβ overproduction (Figure 2C). We further detected whether CRF impaired synaptic function, and found that the presynaptic protein Synapsin I and postsynaptic proteins including PSD95 and GluA1 were reduced (Figure 2D, 2E). These results indicate that CRF promotes AD-like pathologies and synaptic impairments in primary neurons.

We further explored the role of Chk1-CIP2A signaling in inducing AD-like pathologies under stress condition and found CRF treatment resulted in a marked increase in active forms of Chk1 (phosphorylated at Ser317 and Ser345) as well as CIP2A in neurons (Figure 2A, 2B). Based on the previous findings that upregulated ChK1-CIP2A signaling promoted AD pathologies, we demonstrate that CRF could induce DNA damage and activate Chk1-CIP2A signaling pathway, eventually resulting in AD related pathologies and synaptic impairments.

### Chronic stress induces cognitive deficits in mice, which could be prevented by vitamin C and Chk1 inhibitor

**Figure 3:**
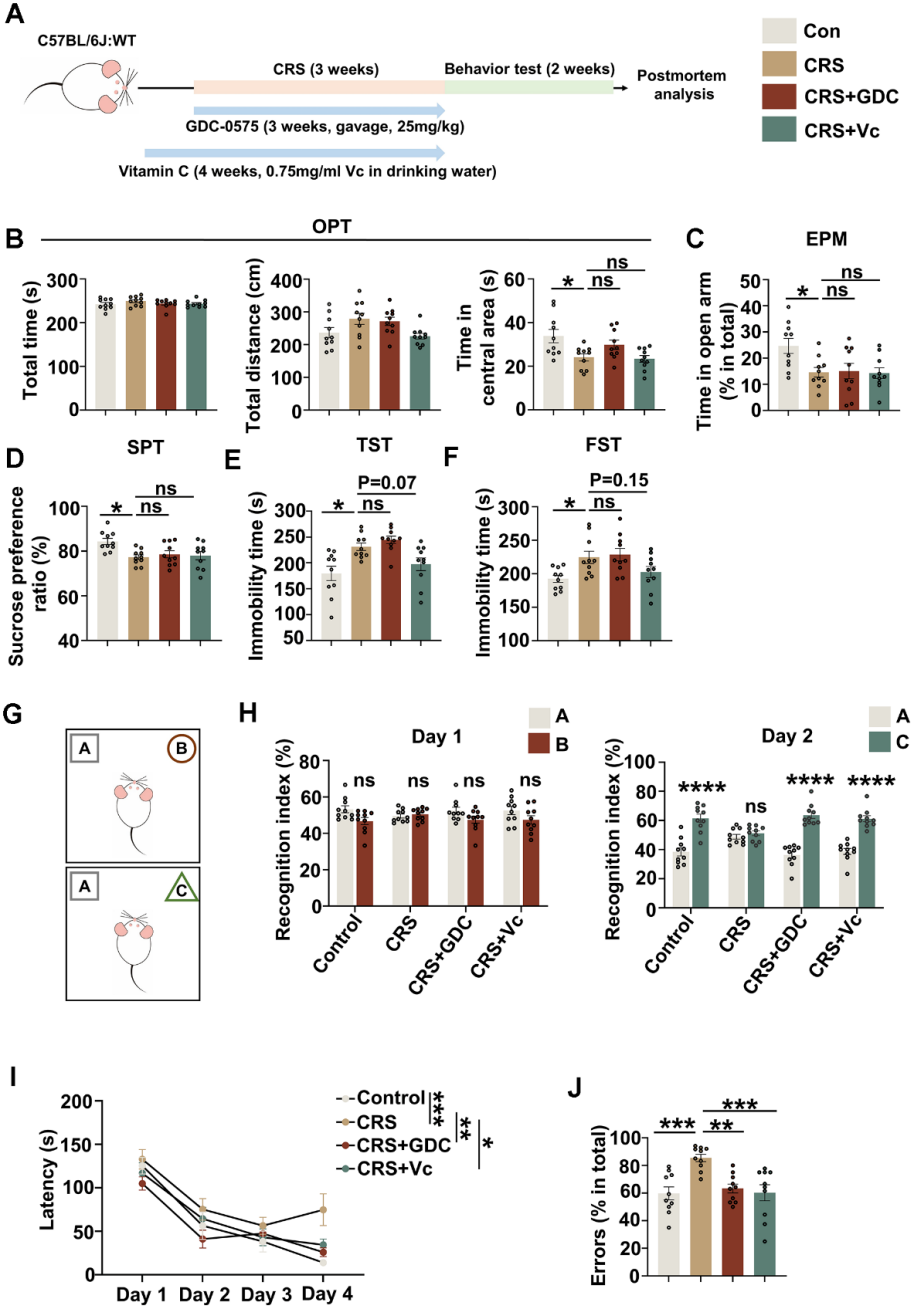
**Chronic stress induces cognitive deficits in mice, which could be prevented by vitamin C and Chk1 inhibitor.** (**A**) Schematic diagram for establishing chronic restraint stress model and treatment with vitamin C or Chk1 inhibitor. (**B**) The total time (s, left), distance (cm, middle) and time in central area (s, right) in open field test. (**C**) The time spending in open arm (%) in elevated plus maze test. (**D**) The sucrose preference ratio (%) in sucrose preference test. (**E**) The immobility time (s) in tail suspension test. (**F**) The immobility time (s) in forced swimming test. (**G**) The experimental design of novel object recognition test. Above is the training trial, below is the testing trial (after 24 hours). (**H**) Left: the recognition index to object A and object B in the training trial; Right: the recognition index to object A and object C in the testing trial. (**I**) The latency of the mice to find the target holes on different training day in Barnes maze test. (**J**) Errors (explore incorrect holes) (% in total) in the probe trial of Barnes maze test. All data represent mean ± SEM, n=10 per group, *P<0.05, **P<0.01, ***P < 0.001, ****P< 0.0001.

Next, we established chronic restraint stress mouse model to further investigate the impact of chronic stress on cognitive function and the role of DNA damage-Chk1-CIP2A signaling pathway in this process. Meanwhile, on the basis of *in vitro* results, we also tried to interfere the possible key pathogenic mechanism through targeting DNA damage and Chk1 activation by vitamin C and Chk1 inhibitor respectively in CRS models. Only male C57BL/6 mice were used because this study is not to examine possible sex differences in the response to chronic restraint stress, but rather to identify the possible mechanism that cause cognitive deficits under this stress. In addition, better molding, less variability [27] and better therapeutic effect [28] may be achieved with male rodents than females. As shown in Figure 3, CRS models were successfully established after 21 consecutive days of restraint. In open field test (OFT), the center time (s) was decreased in CRS group compared with control group, suggesting that CRS group showed anxiety phenotype. The total moving time (s) and distance (cm) showed no difference, indicating neither group had impaired locomotion activity (Figure 3B). Elevated plus-maze test (EPM) was also used to detect anxiety-like behaviors. CRS mice showed decreased tendency of time in open arm (%) (Figure 3C). In sucrose preference test, CRS mice exhibited reduced preference for sucrose compared with control mice (Figure 3D). And immobility time (s) was increased both in tail suspension test (TST) and forced swimming test (FST) (Figure 3E, 3F). These data indicated that CRS mice exhibited depression-like behaviors. In the detection of cognitive function, we found that CRS showed no preference exploring the new subject in novel objective recognition test (NOR) (Figure 3G, 3H), and impaired spatial learning (Figure 3J) and memory (Figure 3I) in Barnes maze test (BMT). Collectively, chronic stress causes both anxiety/depression and cognitive dysfunction in mice.

To prevent the activation of DNA damage-Chk1-CIP2A signaling pathway, we used vitamin C as an antioxidant to protect DNA and Chk1 inhibitor GDC-0575, the latter has been identified to effectively prevent AD-like pathologies and cognitive deficit in APP/PS1 mice through targeting Chk1-CIP2A signaling pathway [23]. Our data showed that both interventions could effectively rescue the cognitive impairment induced by stress in CRS mice (Figure 3H–3J). However, Chk1 inhibitor showed no therapeutic effect on the anxiety and depression of CRS mice; and vitamin C only showed the tendency of rescuing the depression phenotype in CRS mice (Figure 3B–3F). These data suggest that stress might induce anxiety, depression and cognitive deficit through different mechanisms, whereas DNA damage-Chk1 signaling pathway might be more specifically involved in promoting cognitive dysfunction.

### Vitamin C and Chk1 inhibitor reduce DNA damage, Chk1 activation and CIP2A expression, decrease tau phosphorylation and Aβ levels in mice exposed to chronic stress

**Figure 4:**
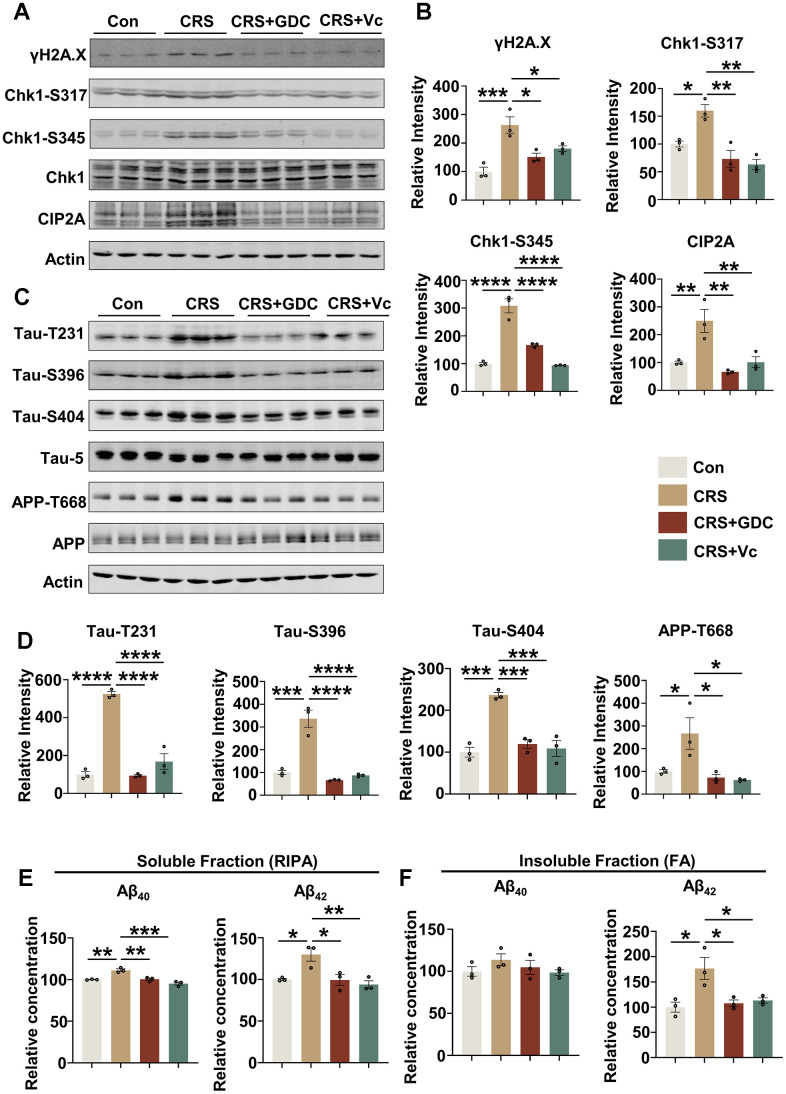
**Vitamin C and Chk1 inhibitor reduce DNA damage, Chk1 activation and CIP2A expression, decrease tau phosphorylation and Aβ levels in hippocampus of mice exposed to chronic stress.** (**A**) Representative immunoblots of γH2A.X, Chk1-pS317, Chk1-pS345, Chk1, CIP2A, β-actin in hippocampal tissues of mice in different groups. (**B**) Quantification of the relative protein levels; non-phosphorylated proteins such as γH2A.X and CIP2A were normalized to the β-actin levels; phosphorylated Chk1-pS317, Chk1-pS345 were normalized to total Chk1. n = 3 per group. (**C**) Representative immunoblots of Tau-pT231, Tau-pS396, Tau-pS404, Tau-5, APP-pT668, APP, β-actin in hippocampus of mice in different groups. (**D**) Quantification of the relative protein expression levels; phosphorylated Tau-pT231, Tau-pS396, Tau-pS404 and APP-pT668 were normalized to Tau-5 and total APP respectively. n = 3 per group. (**E**) The Aβ_40_ and Aβ_42_ in soluble fraction of hippocampal tissues in different groups were detected by ELISA kit. n = 3 per group. (**F**) The Aβ_40_ and Aβ_42_ in insoluble fraction of hippocampal tissues in different groups were detected by ELISA kit. n = 3 per group. All data represent mean ± SEM *P<0.05, **P<0.01, ***P < 0.001, ****P < 0.0001.

To further identify the role of DNA damage-Chk1-CIP2A signaling pathway in cognitive dysfunction caused by stress, we further detected key proteins in this signaling pathway and AD related pathologies. It was found that DNA damage was increased, Chk1 was activated and the expression of CIP2A was elevated in hippocampus and cortex of the brains of CRS mice. These changes were rescued by vitamin C and Chk1 inhibitor (Figure 4A, 4B and Supplementary Figure 3A, 3B). Moreover, vitamin C and Chk1 inhibitor reversed APP and tau hyperphosphorylation, the events downstream of CIP2A upregulation both in hippocampus and cortex in CRS mice (Figure 4C, 4D and Supplementary Figure 3C, 3D). In line with these changes, both treatments decreased Aβ levels in soluble and insoluble fractions of hippocampal tissues from CRS mice (Figure 4E, 4F and Supplementary Figure 3E, 3F). Taken together, these results suggest that chronic stress contributes to AD-like pathologies in which DNA damage-Chk1-CIP2A signaling axis may play an important role.

### Vitamin C and Chk1 inhibitor rescue neuron loss and synaptic impairments in mice exposed to chronic stress

**Figure 5:**
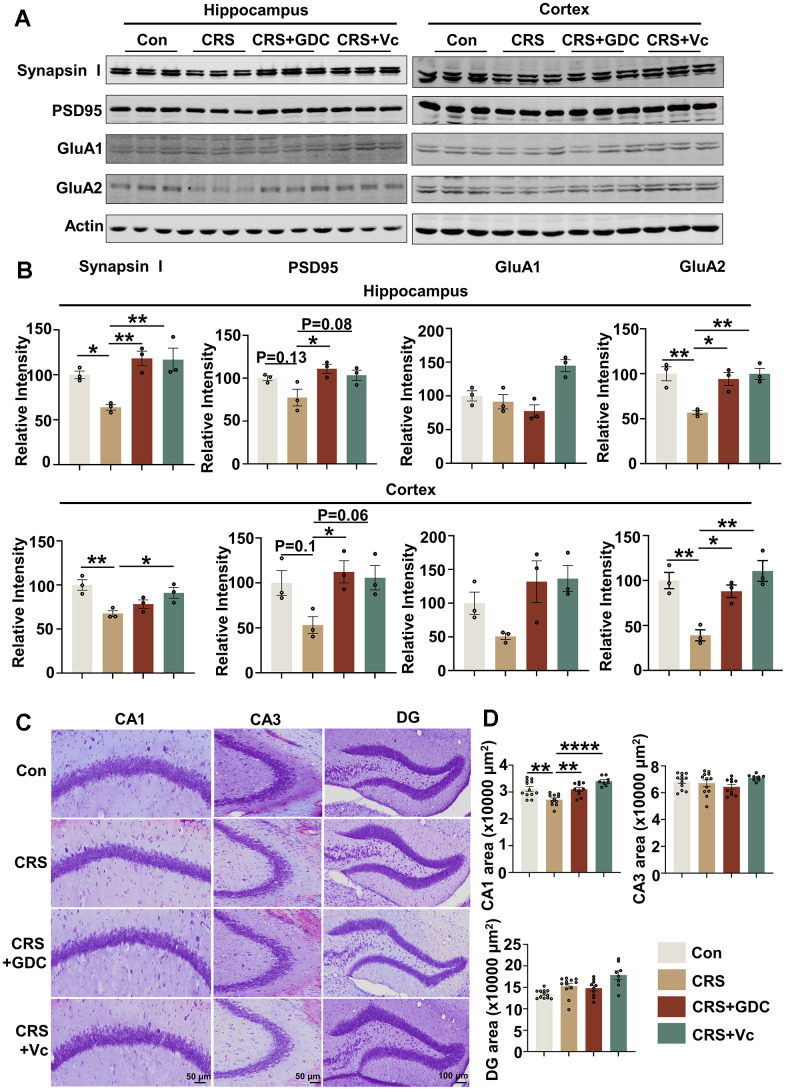
**Vitamin C and Chk1 inhibitor rescue neuron loss and synaptic impairments in mice exposed to chronic stress.** (**A**) Representative immunoblots of Synapsin I, PSD95, GluA1, GluA2, β-actin in hippocampus (left) and cortex (right) of mice in different groups. (**B**) Quantification of the relative protein expression levels, which were normalized to the β-actin levels. n = 3 per group. (**C**) Representative images of Nissl staining in the CA1, CA3 and DG region of mice hippocampus. Scale bar: 50 μm (CA1 and CA3) or 100 μm (DG). (**D**) The quantitative analysis of the area occupied by neurons in the CA1, CA3 and DG region. n = 8-11 per group. All data represent mean ± SEM, *P<0.05, **P<0.01, ****P< 0.0001.

Impaired synaptic plasticity and neuronal damage underlies the cognitive impairment, and we have identified that Chk1 inhibitor has the ability to protect synapses and neurons from the injury of AD pathologies in APP/PS1 mice through reducing Aβ production and tau hyperphosphorylation via downregulating CIP2A [23]. Thus, we evaluated the damage of synapses and neurons in CRS mouse brains and tried to explore whether vitamin C and Chk1 inhibitor exert the same protective effect in this stress model. We detected the levels of pre- and post-synaptic proteins in hippocampal and cortex tissues of the mice in different groups. It was found that the level of presynaptic protein Synapsin I as well as postsynaptic proteins including PSD95 and GluA2 were reduced or showed a tendency of downregulation in CRS mice compared with control mice, and recovered to or even higher than normal levels after supplemented with vitamin C and Chk1 inhibitor (Figure 5A, 5B). Besides, Nissl staining showed neuron loss in CA1 region, but not in CA3 or DG region in hippocampus of CRS mice, was restored by vitamin C and Chk1 inhibitor (Figure 5C, 5D). As mentioned above (Figure 1), neurons in the CA1 region were most vulnerable to DNA damage in hippocampus. This data supports that DNA damage might be an upstream factor of neuronal loss. Taking together, vitamin C and Chk1 inhibitor which inhibit DNA damage-Chk1-CIP2A signaling rescue synaptic impairments and neuronal loss in CRS mice, reinforcing the conclusion that chronic stress causes AD-like cognitive impairment through DNA damage-Chk1-CIP2A signaling.

## DISCUSSION

Stress is a challenging experience in daily life. Under conditions of repeated or prolonged stress, the response would be dysregulated and evoke a series of adverse consequences. Several evidences have identified that stress could induce DNA damage. A study in which plasma from older people with late-life depression (LLD) was examined indicated that the level of 8-hydroxy-2’-deoxyguanosine (8-oxo-dG), a marker of DNA oxidative damage was increased [17]. DNA oxidative damage was significantly elevated in white matter of patients with major depressive disorder (MDD) [18]. In addition to epidemiological evidences, DNA damage was also observed in different chronic stress animal models. DNA damage accumulated in frontal cortex of mice treated with corticosterone [29] or exposed to chronic restraint [19]. In hippocampus of chronic stress animal models, such as CRS [30], CUMS [21] and periodic maternal separation [31], increased oxidative DNA damage and reduced antioxidant enzymes activities were prominent. In the present study, we also found the DNA damage marker γH2A.X was increased in hippocampus and cortex of CUMS and CRS animals. Besides, we reported the cellular specificity of DNA damage under stress conditions, DNA damage mainly occurred in neurons, and was not obvious in astrocytes or microglia. Regional specificity of DNA damage was also uncovered in this study, neurons in CA1 region accumulated severer DNA damage than other brain regions in hippocampus detected in stressed models. Transcriptome analysis has disclosed that neurons in CA1 had higher expression levels of genes related to stress and inflammatory response than in CA3 [32]. Therefore, hippocampal CA1 is a stress-sensitive brain region. This characteristic may explain why CA1 neurons are most vulnerable to cell death under multiple stress conditions, including brain ischemia [33, 34], and psychological distress [35, 36]. Many studies have demonstrated that hippocampal CA1 area is essential to spatial learning and memory [37, 38]. Stimulation of only a small fraction of CA1 pyramidal cells could make significant effects on spatial behavior [39]. Besides, the inactivation of the CA1 neurons could impair delay-dependent object memory [40, 41]. This could explain that CRS mice showed cognitive deficits in NOR and BMT tests.

Several evidences have confirmed that stress is a risk factor for AD. A 30-year cohort study revealed that work-related stress would increase the risk for developing AD (OR=1.55) [42]. Another cohort study indicated that individuals who suffer lifelong work-related stress were at elevated risk of dementia (HR=1.9) and AD (HR=2.2) [43]. In addition to midlife stress, early life stress has been reported to be associated with increased risk of dementia (HR=1.86) [44]. These epidemiological evidences suggest that stress contributes to the development of AD. The underlying mechanisms by which stress leads to AD have also been partially investigated in animal experiments. In many chronic stress models, such as chronic restraint stress [45, 46], chronic social defeat stress [47] and chronic unpredictable mild stress [48, 49], dendritic atrophy, spine loss and reduced expression of neurotrophic factors were detected in animal brains. Normal synaptic function is crucial to learning and memory, thus chronic stress-induced cognitive deficits may be the phenotype of synaptic dysfunction. Moreover, chronic stress caused persistent neuroinflammatory and further induced AD pathogenesis [50, 51]. Besides, chronic stress impaired intestinal mucosal barrier function and caused gut microbiota dysbiosis, and then evoked peripheral and central inflammation, eventually caused memory deficits [52, 53]. Here, we demonstrated a novel molecular mechanism of stress-induced cognitive impairment, based on the previously reported role of DNA damage-ChK1 activation-CIP2A upregulation in promoting AD-like pathologies and cognitive deficit in AD mice models.

We found that 100 nM CRF induced DNA damage, activated Chk1, upregulated CIP2A expression. Meanwhile, CRF caused tau hyperphosphorylation, Aβ overproduction and synaptic impairments in primary neurons. Similar phenomena were observed in brains of mice exposed to chronic restraint stress. Consistent with the significant neuronal loss in hippocampal CA1 region, neurons in this area also showed severer DNA damage. It implies that DNA damage and its downstream pathway may be important risk factors for neuronal loss. In our study, stress hormone CRF rather than CORT was chose to establish cellular stress model for the following reasons: First, the purpose of our experiment is to investigate the underlying mechanism by which stress-induced DNA damage cause AD pathogenesis. However, CORT could not induce DNA damage at a low concentration without cytotoxicity. Even though 300 or 400 μM CORT caused DNA damage, they also evoked significant cellular toxicity. On the contrary, CRF at low concentration (100 nM) could cause DNA damage without inducing significant cytotoxicity. Second, previous study has reported that the phosphorylation levels of Chk1 were significantly increased in hippocampal neurons after CRF treatment [54]. In our study, we confirmed the Chk1 activation in response to CRF stimulation and DNA damage, and further observed CIP2A upregulation and typical AD-like pathologies including tau hyperphosphorylation, Aβ overexpression and synaptic degeneration in CRF-treated neurons. Similar changes, such as tau hyperphosphorylation and increased Aβ levels were also observed in CRF-overexpressing mice, and corticotropin-releasing factor receptor (CRFR) 1 antagonist could attenuate AD related pathologies [55, 56]. Taking together, these data support the hypothesis that stress hormone CRF may promote AD pathogenesis through DNA damage-Chk1-CIP2A signaling pathway.

This molecular mechanism underlying stress-induced cognitive dysfunction was further confirmed by rescuing experiments using Chk1 inhibitor and DNA protector in stressed mice. These mice showed both learning and memory impairment and emotional problems such as anxiety and depression. For targeting DNA damage-Chk1-CIP2A signaling pathway, we used GDC-0575, a second-generation Chk1 inhibitor which is less toxic, more potent and orally effective compared to the first-generation inhibitors. Our previous study confirmed that this drug could reduce tau hyperphosphorylation and Aβ production in AD cell models and APP/PS1 mice [23]. We also used vitamin C as an antioxidant to prevent the oxidative DNA damage as the second intervention. A study has revealed that vitamin C administration restored the increased oxidative DNA damage and apoptosis in potassium dichromate-exposed rats [57]. Besides, vitamin C attenuated oxidative DNA damage induced by surgery in APP/PS1 mice [58]. In our study, both GDC-0575 and vitamin C efficiently restored the activation of Chk1, CIP2A overexpression, tau/APP hyperphosphorylation and cognitive deficits in CRS mice, confirming that CIP2A upregulation-tau/APP hyperphosphorylation, and cognitive impairment is downstream of DNA damage-Chk1 activation in stressed mice. Meanwhile, we observed that Chk1 inhibition could only prevent the occurrence of cognitive dysfunction with no effect on rescuing the anxiety/depression phenotypes of the CRS mice. These data suggest that Chk1 activation plays a unique role in promoting AD-like cognitive deficit. Compared with Chk1 inhibitor, vitamin C showed a general and broader protective effect on the neurologic function. Several clinical and experimental data has confirmed that vitamin C have anxiolytic and antidepressant effect [59, 60]. In our study, the anxiety of the CRS mice was not rescued by vitamin C. However, there was a trend for vitamin C to ameliorate the depressive-like behaviors, although there was no significant difference. The possible reasons are suspected as follows: First, the dose and duration of vitamin C administrated in our study may be different from other studies; Second, the abilities of vitamin C to rescue anxiety- or depression-like behaviors depend on the severity of anxiety or depression, which may vary in different experiments [59]. Finally, neuronal circuits or molecular mechanisms underlying anxiety and depression are different, vitamin C may relatively specifically target the signaling pathway of depression, which need further exploration.

In conclusion, DNA damage-Chk1-CIP2A signaling pathway participates in stress-induced AD-like pathologies and cognitive deficit. Vitamin C and Chk1 inhibitor rescue AD related pathologies under stress condition. Our research discloses a new mechanism of stress-induced cognitive impairment, and provide valuable preventive and therapeutic options for AD.

## MATERIALS AND METHODS

### Antibodies and reagents

The primary antibodies used in this research were as follows: anti-γH2A.X (ab26350, Abcam; 80312S, Cell Signaling Technology), anti-NeuN (26975-1-AP, Proteintech), anti-GFAP (12389S, Cell Signaling Technology), anti-Iba1 (ab178846, Abcam), anti-Chk1-pS317 (12302S, Cell Signaling Technology), anti-Chk1-pS345 (39233, Gene Tex; A21009, Abclonal), anti-Chk1 (A7653, Abclonal), anti-CIP2A (A12267, Abclonal), anti-Tau-pT231 (R381181, Zen-Bioscience), anti-Tau-pS396 (381213, Zen-Bioscience), anti-Tau-pS404 (11112, SAB), Tau-5 (ab80579, Abcam), anti-APP-pT668 (6986S, Cell Signaling Technology), anti-APP (60,342–1-Ig, Proteintech), anti-PSD95 (36233S, Cell Signaling Technology; 30255-1-AP, Proteintech), anti-Synapsin- I (5297S, Cell Signaling Technology), anti-GluA1 (13185S, Cell Signaling Technology), anti-GluA2 (13607S, Cell Signaling Technology), anti-β-actin (AC026, Abclonal). The second antibodies for Western blotting were purchased from LICOR-Biosciences (Cat#926-32210, IRDye-800CW Goat anti-Mouse IgG Secondary Antibody; Cat#926-32211, IRDye-800CW Goat anti-Rabbit IgG Secondary Antibody). The second antibodies for immunofluorescence were obtained from Proteintech (Cat#SA00013-1, CoraLite488-conjugated Goat Anti-Mouse IgG; Cat#SA00013-4, CoraLite594-conjugated Goat Anti-Rabbit IgG). Corticosterone (Cat# HY-B1618), Corticotropin-releasing factor (human) (Cat# HY-P0086) and GDC-0575 (Cat#HY-112167) were from MedChemExpress, USA. Vitamin C (Cat# 255564) was purchased from Sigma-Aldrich, USA.

### Animals and treatment

C57B6/L mice (male, 8 weeks old, 22 ± 0.5 g) were supplied by Liaoning Changsheng biotechnology. Sprague-Dalley rats (female, 8 weeks old, 250 ± 25 g) were obtained from the Experimental Animal Central of Disease Control and Prevention Center of Hubei Province. All animals were housed under a regular 12/12 hours light/dark cycle, appropriate temperature (22 ± 2° C) and humidity (55 ± 15%) with unrestricted access to food and water.

Mice were randomly divided into four groups: the control group (Con), the chronic restraint stress group (CRS), the CRS treated with Chk1 inhibitor (GDC-0575) group (CRS+GDC) and the CRS treated with vitamin C group (CRS+Vc). GDC-0575 was dissolved in 10% DMSO (25 mg/ml), 40% PEG300, 5% Tween-80, and 45% saline and mixed to obtain a final concentration of 3.75 mg/ml before every gavage. The CRS+GDC group were orally administered with GDC-0575 (25 mg/kg) in 6 days of 3 weeks. Other mice were treated by the corresponding solvent. The vitamin C were solubilized in distilled water and the CRS+Vc group were provided with 0.75 g/ml vitamin C in drinking water one week prior to chronic restraint stress for 4 weeks, while others received normal drinking water. Because vitamin C is easily to be degraded, the solutions were prepared fresh daily.

Mice exposed to chronic restraint stress were put into a well-ventilated 50 mL centrifuge tube to restrain movements for 3 hours (10:00 to 13:00) per day for 21 consecutive days. The acute restraint stress group were limited in a same tube for three hours and sacrificed immediately following stress.

The rats exposed to chronic unpredictable mild stress were given the following stressors: (1) electrical shock to the feet (2 seconds, 0.5 mA, 5 times in 10 minutes); (2) cold water bath (20 minutes); (3) water deprivation (12 hours); (4) food deprivation (24 hours); (5) reversed day/night light cycle (24 hours); (6) physical restraint (2 hours); (7) low temperature (4° C, 2 hours); (8) high temperature (45° C, 20 minutes); (9) Cage tilting (45° angle, 24 hours); (10) tail pinch (90 seconds); (11) crowed living condition (12 hours); (12) moist bedding (24 hours); (13) horizontal vibration (100 rpm, 20 minutes). A combination of 0-3 kinds of stressors were randomly applied each day and the same stressor was not used continuously within 2 days during the period of 28 days.

### Behavioral tests

### Open field test (OFT)

The test was performed in an empty area (50 cm × 50 cm × 50 cm plastic container, Techman Software Co, Ltd, Chengdu, China). The area was divided into 25 equal squares (5 × 5 sectors), the middle 3 × 3 sectors were denoted as central area, and others were peripheral areas. Mice were placed in the center at the beginning of test and allowed to explore freely for 5 minutes. The time and distance in total and in central area were recorded. The total time and distance were used to assess to locomotor activity. The center duration (%) were calculated to measure anxiety-like behavior.

### Elevated plus-maze test (EPM)

Mice were placed in the center of an apparatus with two open arms and two closed arms for 5 minutes (25 cm long, 6 cm wide). The device was elevated 50 cm. Software measured the time spent on the open arms and the number of entries into open and closed arms. The time in open arms (%) were calculated to assess anxiety-like behavior.

### Sucrose preference test (SPT)

Sucrose preference test was used to assess depression-like behavior. Mice were placed individually into a cage and given with two identical bottles containing 2% sucrose solution or pure water, respectively for 12 hours. On the second period, the two bottles were changed. Next, mice were given water deprivation for 12 hours. Finally, each mouse was provided with the two bottles mentioned above. After 24 hours, we recorded the consumption of solution in each bottle. The sucrose preference was calculated by the formula: Sucrose preference (%) = [(sucrose consumption) / (water consumption + sucrose consumption)] × 100%.

### Tail suspension test (TST)

The tail of each mouse was fixed with adhesive plaster and suspended for 5 minutes. The head was 15 cm above the ground. The immobility and struggling time were recorded during the testing period. More immobility time was considered as depression-like behavior.

### Forced swimming test (FST)

Mice were arranged in an open cylinder container (25 cm high×18 cm diameter) filled with fresh water (25 ± 2° C) to 15 cm high and kept the head above the water to breath. The duration of immobility was scored during the 5-min session to assess depression-like behavior.

### Novel object recognition test (NOR)

The device was a 50 cm × 50 cm × 50 cm plastic box. Before the test, mice allowed to move freely in this container to adapt experiment. On the first day of training trial, object A and object B were placed on two corners in this box to allow mice to explore the two objects freely for 5 minutes. The exploring time on object A and B were recorded, denoted as TA and TB. On the second day of testing trial, the object B was replaced with another new object C, and the three objects have different color and shape. Mice were returned to the box for 5 minutes to move freely. The time spent on exploring object A and C were measured, denoted as TA and TC. The recognition index (%) was expressed as follows: The first day is TA/ (TA + TB), TB/ (TA + TB), the second day is TA/ (TA + TC), TC/ (TA + TC).

### Barnes maze test (BMT)

The test was conducted in a 90 cm-diameter circular plate with 20 round holes (5 cm in diameter) on the edge. The test comprised two steps: training (4 days) and probe (1 day).

During the training trial, a hole was set as escape box (a square box with 10 cm × 10 cm × 6 cm under this hole), where mice could stay away from light. Mice were limited in a cylindrical cage at the center of the device for 5 seconds at the beginning of training. Then, mice were released to explore the area, find and enter the escape box within 3 minutes. If mice did not enter the box, the experimenter guided them into this box at the end of this session. And mice remained for 30 seconds after reaching the escape box. Each mouse was trained for three trails per day, and an interval of less than two hours was guaranteed between adjacent training sessions. In the probe trial, the escape box was removed and mice were allowed to explore the area for 90 seconds. The escape latency (the time until reaching the escape box) during training session and the number of errors (explore incorrect holes) in the probe test were recorded.

### Primary neuron culture and treatment

Primary cortical neurons were isolated from E16-E18 C57B6/L mice. Cortical tissues were dissociated, and digested by trypsin for 15 minutes, followed by adding medium containing DMEM/F12 with 10% fetal bovine serum to terminate the digestion. Then cells were suspended again and plated onto 6-well plates coated with poly-D-lysine after suspension and cultured in an incubator (5% CO_2_, 37° C). After 6 hours, medium was replaced with neurobasal medium supplemented with 2% B-27, 1% GlutaMAX (2 mM), 1% penicillin (50 U/mL), and streptomycin (50 μg/ mL). The medium was half-changed every 3 days during the culture. After 9 days, cells were treated with CORT and CRF at different concentrations for 24 hours. Then the neurons and culture media were harvested for following tests.

### Western blotting

Cultured neurons and brain tissue were lysed with RIPA lysis buffer (Beyotime Biotechnology, Shanghai, China) containing PMSF (1:100) and proteinase inhibitor cocktail (200 mM AEBSF, 30 μM aprotinin, 13 mM bestatin, 1.4 mM E64, and 1 mM leupeptin in DMSO, 1:100) (Yeasen Biotech, Shanghai, China Cat#20124ES03). Then cell lysates or tissue homogenates were added with the loading buffer (final concentration: 50 mM Tris-HCl, pH 7.6, 2% SDS, 10% glycerol, 10 mM DTT, and 0.2% bromphenol blue) and boiled at 100° C for 10 minutes. Next, samples were centrifuged at 12,000 *g* at 4° C for 10 minutes and sonicated. Finally, the concentration of protein was measured by BCA kit (Beyotime Biotechnology, Shanghai, China).

Protein was electrophoresed in 10% or 12% SDS-polyacrylamide gel and then transferred onto 0.45 μm nitrocellulose membranes (Cytiva, USA). After blocking with 5% non-fat milk for 1 hour, the membranes were incubated overnight at 4° C with primary antibodies. Then they were washed 3 times for 10 minutes each by TBST and incubated with secondary antibodies at room temperature for 1 hour. Finally, these blots were washed again as above and visualized using Odyssey Infrared Imaging System (LICOR Biosciences, USA). The intensity of the protein bands was quantitatively analyzed by Image J software (Rawak Software, Inc.).

### LDH cytotoxicity assay

The LDH cytotoxicity assay was performed according to the manufacturer’s procedure (Cat# C0017, Beyotime Biotechnology, Shanghai, China).

### ELISA assay

The culture media of primary neurons was collected for ELISA assay directly. For detecting the Aβ levels in cell lysates and brain tissues, cell and brain samples were lyzed in PBS (containing 1:100 PMSF and 1:100 protease inhibitor cocktail) and centrifuged for 10 minutes at 12,000 g at 4° C. The supernatant was RIPA-soluble fraction. The pellets obtained from brain tissues were further dissolved in 70% formic acid as the RIPA-insoluble fraction. The levels of Aβ_40_ and Aβ_42_ were detected according to the manufacturer’s instructions (Elabscience Biotechnology, Wuhan, China).

### Fluorescence imaging and confocal microscopy

The mice were anesthetized by isoflurane, perfused with 0.9% normal saline and 4% paraformaldehyde (PFA) intracardially. Then the brain was removed and further fixed with 4% PFA at 4° C. After 48 hours, these brains were immersed in 30% sucrose in PB until they plunged to the bottom. Then the brains were embedded in OCT (optimum cutting temperature compound, Sakura, USA), frozen, and sectioned at 30 μm using a cryotome (CM1950, Leica, Germany). Brain slices were washed by PBS three times and permeabilized in 0.5% Triton X-100 for 20 minutes, then incubated with 5% bovine serum albumin. After blocking, primary antibodies, secondary antibodies and DAPI (Beyotime Biotechnology, Shanghai, China) were used in succession. All the images were collected by confocal microscope (LSM780, Zeiss, Germany).

### Nissl staining

Brain slices prepared as above were stained according to the manufacturer’s procedure (Leagene Biotechnology, Beijing, China) and images were collected under the microscope (VS120, Olympus, Japan). The area occupied by neurons in the hippocampal CA1, CA3 or DG regions were measured by Image J software.

### Statistical analyses

Data were analyzed as the mean ± SEM and analyzed using GraphPad Prism 9 software (GraphPad Software, Inc., La Jolla, CA). The one-way analysis of variance (ANOVA) with Tukey’s post hoc test was used to compare the differences among groups. And Student’s test was used to determine between two groups. The significance was set at *P* < 0.05, and the test was two-tailed.

### Data availability

The data that support the findings of this study are available from the corresponding author upon reasonable request.

## Supplementary Material

Supplementary Figures
