# Supplementary material for: Chronic stress induces Alzheimer’s disease-like pathologies through DNA damage-Chk1-CIP2A signaling
Source: Aging (Albany NY). 2024 May 30;16(10):9168–87. doi: 10.18632/aging.205862 (PMC11164505; doi:10.18632/aging.205862)
Supplement: Supplementary Figures [file aging-16-205862-s001.pdf]

SUPPLEMENTARY FIGURES

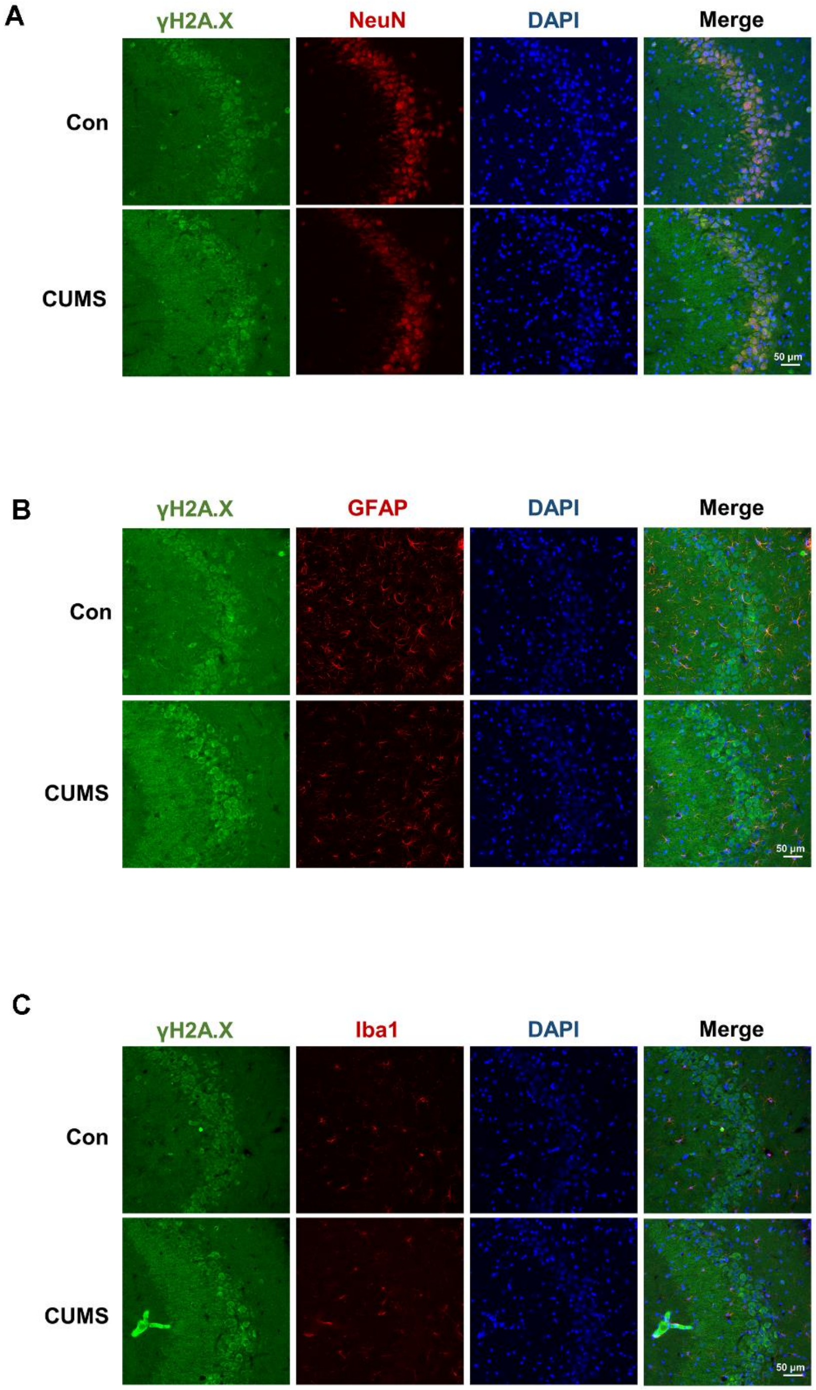

Supplementary Figure 1. DNA damage mainly occurs in neurons, not in astrocytes or microglia. (**A**) Representative fluorescence images of γH2A.X (green), NeuN (red) and DAPI (blue) in CA3 region of hippocampus in CUMS rats. Scale bar: 50 µm. (**B**) Representative fluorescence images of γH2A.X (green), GFAP (red) and DAPI (blue) in CA3 region of hippocampus in CUMS rats. Scale bar: 50 µm. (**C**) Representative fluorescence images of γH2A.X (green), Iba1 (red) and DAPI (blue) in CA3 region of hippocampus in CUMS rats. Scale bar: 50 µm.

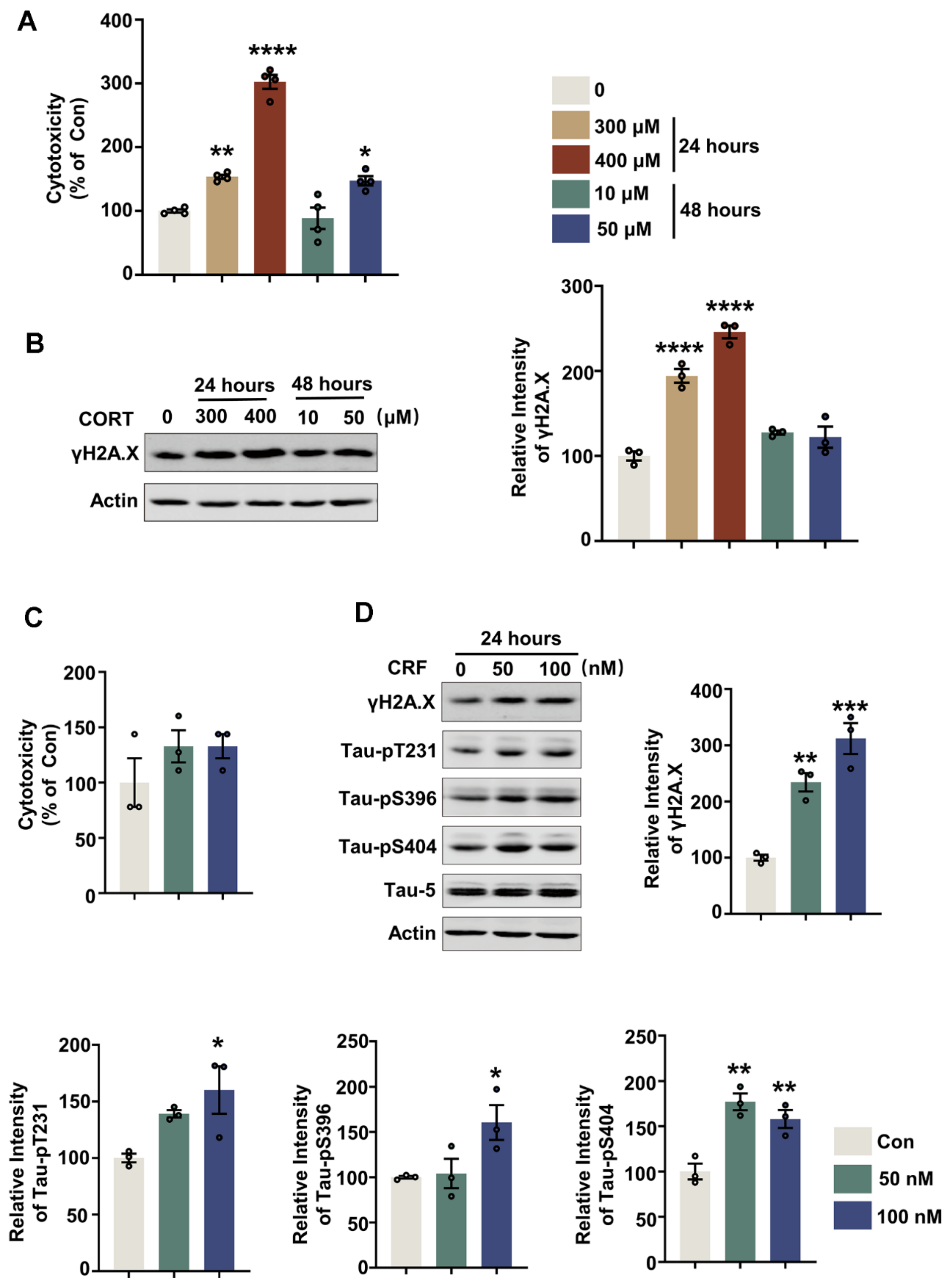

Supplementary Figure 2. CRF and CORT induce DNA damage in primary neurons. (**A**, **B**) Primary neurons were treated with 300 or 400 μM corticosterone (CORT) for 24 hours, 10 or 50 μM CORT for 48 hours. (**A**) LDH cytotoxicity assay results of the cells treated with CORT. n = 4 per group. (**B**) Representative immunoblots and quantification analysis of γH2A.X (normalized to the β-actin levels). n = 3 per group. (**C**, **D**) Primary neurons were treated with 50 or 100 nM CRF for 24 hours. (**C**) LDH cytotoxicity assay of the cells treated with CRF. n = 3 per group. (**D**) Representative immunoblots of γH2A.X, Tau-pT231, Tau-pS396, Tau-pS404, Tau-5 (total tau), and β-actin. And quantification analysis of γH2A.X (normalized to the β-actin levels), Tau-pT231, Tau-pS396, Tau-pS404 (normalized to the Tau-5 levels). n = 3 per group. All data represent mean ± SEM, **P*<0.05, ***P*<0.01, ****P* < 0.001, *****P*< 0.0001.

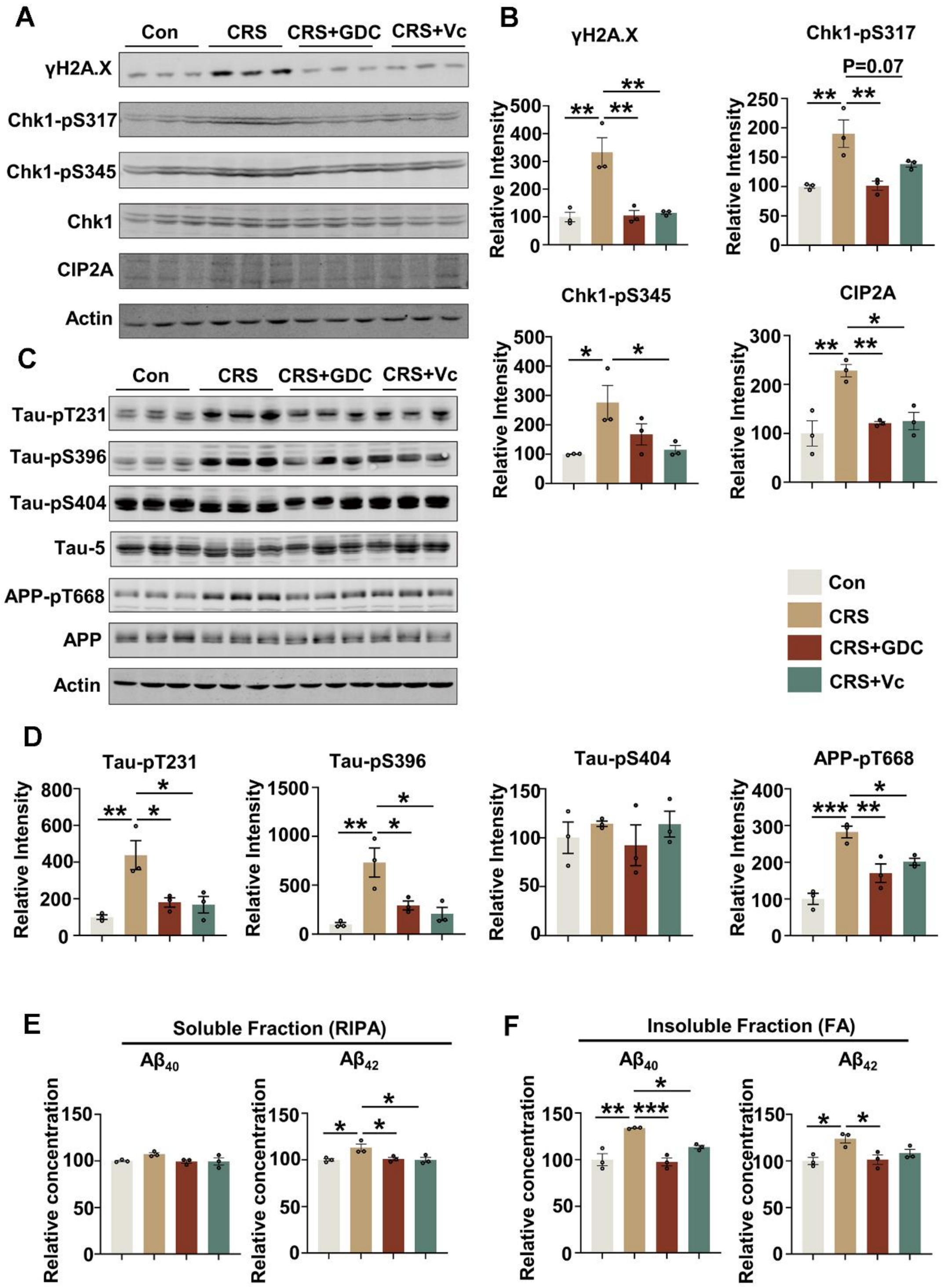

Supplementary Figure 3. Vitamin C and Chk1 inhibitor reduce DNA damage, Chk1 activation and CIP2A expression, decrease tau phosphorylation and Aβ levels in cortex of mice exposed to chronic stress. (**A**) Representative immunoblots of γH2A.X, Chk1-pS317, Chk1-pS345, Chk1, CIP2A, β-actin in cortex of mice in different groups. (**B**) Quantification of the relative protein expression levels; non-phosphorylated proteins such as γH2A.X and CIP2A were normalized to the β-actin levels; phosphorylated Chk1-pS317, Chk1-pS345 were normalized to total Chk1. n = 3 per group. (**C**) Representative immunoblots of Tau-pT231, Tau-pS396, Tau-pS404, Tau-5, APP-pT668, and APP, β-actin in cortex of mice in different groups. (**D**) Quantification of the relative protein expression levels; phosphorylated Tau-pT231, Tau-pS396, Tau-pS404 and APP-pT668 were normalized to Tau-5 and total APP respectively. n = 3 per group. (**E**) The $A\beta_{40}$ and $A\beta_{42}$ in soluble fraction of cortex tissues in different groups were detected by ELISA kit. n = 3 per group. (**F**) The $A\beta_{40}$ and $A\beta_{42}$ in insoluble fraction of cortex tissues in different groups were detected by ELISA kit. n = 3 per group. All data represent mean ± SEM, **P*<0.05, ***P*<0.01, ****P* < 0.001.
